# New perspectives in functional hypogonadotropic hypogonadism: beyond late onset hypogonadism

**DOI:** 10.3389/fendo.2023.1184530

**Published:** 2023-06-29

**Authors:** Matteo Spaziani, Francesco Carlomagno, Chiara Tarantino, Francesco Angelini, Ludovica Vincenzi, Daniele Gianfrilli

**Affiliations:** ^1^ Section of Medical Pathophysiology and Endocrinology, Department of Experimental Medicine, Sapienza University of Rome, Rome, Italy; ^2^ Centre for Rare Diseases (Endo-ERN Accredited), Policlinico Umberto I, Rome, Italy

**Keywords:** functional hypogonadotropic hypogonadism, diabetes, obesity, drugs, HIV, low energy availability, gonadotropins, late onset hypogonadism

## Abstract

Functional hypogonadotropic hypogonadism (FHH) is an increasingly frequent condition, whose pathological mechanisms are not yet fully clarified. The concept of FHH has now completely replaced that of late onset hypogonadism, that only concerned the ageing man. FHH is the result of an impairment of the hypothalamic-pituitary gonadal axis (HPG-A) function, resulting in decreased testosterone concentrations associated with low or inappropriately normal gonadotropin levels and infertility; it can be diagnosed once organic causes of hypogonadism are excluded. The growing occurrence of FHH derives from its association with widespread conditions, such as obesity and diabetes mellitus, but also to the increasing ease and frequency of use of several drugs, such as opioids, glucocorticoids, and sex steroids. Moreover, given the tendency of many subjects to excessive physical activity and drastic reduction in caloric intake, FHH may also be secondary to low energy availability. Finally, the association with HIV infection should not be overlooked. Therefore, there is an important variability in the diseases that can lead to FHH. Despite the heterogeneity of the underlying pathologies, the mechanisms leading to FHH would seem quite similar, with the initial event represented by the impairment at the HPG-A level. Nevertheless, many different biological pathways are involved in the pathogenesis of FHH, therefore the aim of the current paper is to provide an overview of the main relevant mechanisms, through a detailed analysis of the literature, focusing specifically on pathogenesis and clinical, diagnostic and therapeutic aspects.

## Introduction

For a long time the functional form of hypogonadotropic hypogonadism coincided with the concept of late-onset hypogonadism, which was defined as a clinical and biochemical syndrome associated with advancing age and testosterone deficiency (1). A new classification emerged in the last few years, based on the concept of organic opposed to functional hypogonadism. Specifically, functional hypogonadotropic hypogonadism (FHH) is defined as the coexistence of androgen deficiency-like features and low serum testosterone concentrations, occurring in the absence of both intrinsic structural hypothalamic-pituitary-gonad axis (HPG-A) pathology and of specific pathologic conditions suppressing the HPG-A (2).

FHH derives from a hypothalamic or pituitary dysfunction, that results in a decreased testosterone production associated with low or inappropriately normal LH levels and infertility (3). As known, testosterone appears as an essential hormone for testicular development and ultimately spermatogenesis. Indeed, testosterone is involved in Sertoli cell development, and its role in supporting spermatogenesis is indirectly highlighted by the halting of spermatogenesis during meiosis in case of impaired testosterone signaling (4).

It may be the consequence of several comorbidities such as obesity, diabetes mellitus and low energy availability (LEA), subordinate to nutritional deficit with or without excessive expenditure. It may also be secondary to drugs, such as opioids, sex steroids, GnRH analogues, and glucocorticoids. Each of these conditions influences the HPG-A function with different mechanisms, which are not completely understood. For example, in FHH associated with low energy expenditure (LEA), the pathophysiology is strictly functional, and although a role for prolactin and cortisol has also been hypothesized, the specific pathogenesis has not yet been fully elucidated (5–7), whereas drugs most commonly act directly on the HPG-A at the central level. This review also explores the complex interactions between the HPG-A and obesity and/or dysglycemia, where the role of leptin, estradiol, adipose tissue, and inflammation has also been addressed.

Moreover, we also analyzed the potential management of each condition associated with FHH. Treatment represents a hot topic of discussion, since FHH can be reversible when the underlying causes are removed. Nonetheless, there is a significant lack of high-quality evidence and clinical trials in this field. Lifestyle modifications play a key role in the management of FHH due to obesity and diabetes, but also in the spectrum of LEA (8–10). In this regard, it is essential to reinforce the concept that the first line of treatment is to resolve the underlying condition of FHH; however, the need for testosterone replacement therapy (TRT) is not uncommon, and in some conditions, such as obesity or HIV infection, when the attempts to solve the underlying condition have failed, TRT could be useful in addressing the disease and improving hypogonadal symptoms (11, 12). However, the potential negative effect of exogenous TRT on spermatogenesis should not be underestimated; a careful evaluation of the subject to be treated should therefore be carried out. Finally, the minimum effective TRT dose in inducing a substantial improvement in symptoms should be chosen (13). Finally, for the management of infertility, the treatment with exogenous gonadotropins may also be considered (2). In this regard, such treatment has already been widely and successfully used in other forms of HH, such as congenital HH, in which it may be useful also for pubertal induction, allowing a physiological maturation of the testis, which would obviously not be possible with the use of TRT (14).

The aim of this paper is to address the main aspects related to FHH, focusing on the pathophysiologic, clinical and therapeutic tools associated with the main causes of FHH.

**Figure 1:**
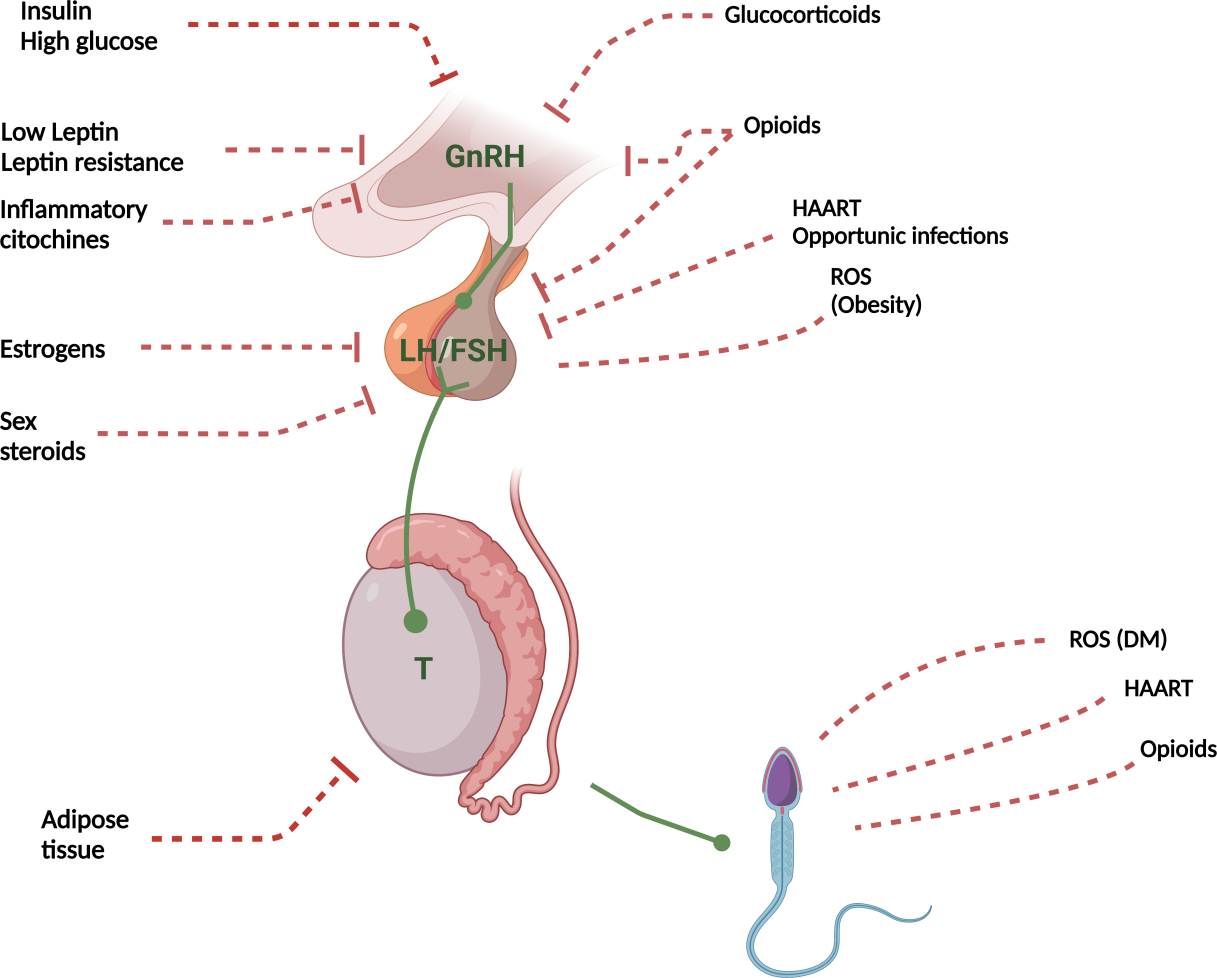
Pathophysiological mechanisms of FHH. T, Testosterone; ROS, Reactive oxygen species; DM, Diabetes Mellitus; HAART, Highly Active AntiRetroviral Therapy. Red dashed lines with lock, Inhibition; Red dashed lines, Indirect damage; Green arrows, Activation.

**Figure 1** summarizes the main pathophysiological mechanisms involved in the genesis of FHH.

## Diabetes

Diabetes mellitus, both type 2 (T2DM) and 1 (T1DM), represents one of the most important determinants of FHH and infertility. A large amount of studies shows that diabetes, particularly if uncontrolled, can lead to infertility in both sexes through pathophysiologic mechanisms acting at several levels, such as alterations in gonadal structure (degenerative and apoptotic changes), HPG hormonal synthesis, or sexual dysfunction (15, 16).

Insulin-resistance plays a major role in the pathogenesis of T2DM, and the resulting hyperinsulinism may be crucial in the development of FHH. In the brain, insulin receptors mediate action of GnRH secreting anterior preoptic hypothalamic neurons; therefore, the occurrence of insulin-resistance can cause an impaired secretion of GnRH, leading to a reduction of LH and testosterone; moreover, a study on knockout mice with a specific deletion of the brain insulin receptor demonstrated HH and infertility (17). High glucose levels may also determine specific toxicity in GnRH-secreting neurons, as seen in women with T1DM, who display an inhibitory effect of hyperglycemia on the hypothalamic secretion of GnRH, causing amenorrhea (17, 18).

The female gonad is subject to significant alterations secondary to hyperinsulinism, as seen in polycystic ovary syndrome (PCOS), since the ovaries express insulin receptors in the granulosa, theca, and stromal compartments. In addition, insulin also binds to the ovarian (as well as testicular) Insulin Growth Factor-1 (IGF1) receptor (19), which causes the classic PCOS increase in androgen production and follicle stimulation in granulosa and theca cells (20, 21). The same issues can arise in T1DM, caused by the exogenous hyperinsulinemia due to intensive glucose control regimens (22).

Studies on rats have shown that insulin is also secreted by both the testes and sperm cells (22, 23), and analogous papers have shown that insulin therapy can re-establish both sperm count and motility, most likely by restoring the function of the HPG-A (24, 25).

It should not be forgotten that T2DM could be one of the manifestations of the so-called hidden hypercortisolism (subclinical hypercortisolism), a condition of biochemical cortisol excess without the clinical features specific to Cushing syndrome. These subjects are at greater risk of experiencing certain comorbidities, such as hypertension, increased risk of fragility fractures and T2DM, although the diagnosis is far from easy. High cortisol levels together with hyperglycemia can be detrimental to gonadal function, making hidden hypercortisolism a further condition to consider as a possible cause of FHH (26).

The assessment of the HPG-A hormone pattern and semen parameters in men with T1DM and T2DM has been carried out in an extremely limited number of studies, with relatively small and highly heterogeneous cohorts (27, 28). Few studies focused on T1DM, some of them finding no significant differences (27, 29), whereas three found partly conflicting results: significantly higher levels of gonadotropins without differences in testosterone (27), lower LH levels in patients with uncontrolled diabetes (30) and significantly lower testosterone concentrations without differences in gonadotropins (27). T2DM studies also yielded mixed and poorly reproducible results (28, 31). Two meta-analyses on this topic underlined the low power of the available data (32, 33).

An interesting paper made it clearer to determine the influence of diabetes on seminal parameters (especially the decrease in sperm progressive motility), showing how the pathophysiological mechanisms of damage may be different in T1DM and T2DM. A higher oxidative stress is present in T2DM patients, with an increased concentration of seminal fluid leukocytes secondary to an amicrobial inflammatory condition, resulting in decreased sperm vitality and increased sperm DNA fragmentation. On the contrary, in T1DM patients the damage is most prominently based on the presence of mitochondrial damage caused by a lower degree oxidative damage than in T2DM subjects, associated with an altered epididymal voiding, leading to low ejaculate volume (34).

Further studies are needed to better describe the hormonal and gonadal consequences of diabetes, although a proper lifestyle, combined with adequate pharmacological control, represent the basis for both reducing cardiovascular risk and preserving reproductive health. Notably, with regard to treatment, the European Academy of Andrology (EAA) recommends against TRT with the specific intent to improve glyco-metabolic control in men with T2DM and/or metabolic syndrome, however its use is recommended in the case of symptomatic FHH (2).

## Obesity

The escalating epidemic of overweight and obesity has important clinical implications since excessive adiposity can progressively cause and/or exacerbate a wide spectrum of co-morbidities, including metabolic syndrome and cardiovascular disease, as well as constituting the most common form of FHH (35, 36). The prevalence of hypogonadism in subjects with severe obesity (BMI > 40 kg/m^2^) is up to 75%, compared to that in normal weight adult males which ranges from 2 to 39% (37–39).

Men who are overweight and obese tend to have lower concentrations of sex hormone-binding globulin (SHBG) compared to lean subjects, which affect free testosterone levels (40). SHBG is a glycoprotein that binds sex hormones, inhibiting their biological activity, and has a pivotal role in the crosstalk between metabolic disorders and testosterone deficiency. As a matter of fact, visceral adiposity is negatively associated with SHBG levels, and the possible coexistence with hyperinsulinemia may reduce the liver production of SHBG (41). Moreover, much evidence attests that SHBG is down-regulated by pro-inflammatory cytokines, whose levels are considerably increased in obese patients (42). This reduction could result in a temporary increase of free testosterone levels, which might raise aromatase activity even further, accentuating the conversion of testosterone to estradiol. These modifications result in negative feedback on the HPG-A, eventually leading to a reduction of both total and free testosterone levels, combined with low SHBG levels (43). Waist circumference and BMI are also associated with the degree of HPG-A dysfunction (44–46). In addition, hypogonadism is associated with fat accumulation, leading to a vicious cycle in which abnormal adipose tissue (AT) expansion affects testosterone production, resulting in further accumulation of AT (47).

AT plays a critical role in the development of FHH via both direct and indirect mechanisms. Directly, it induces androgen deprivation both by sequestering testosterone from the systemic circulation, and through its conversion to estradiol (48–50). Obese subjects, in fact, have an increased aromatase activity, responsible for testosterone conversion into estrogens. On the other hand, increased estrogen levels reduce LH pulse amplitude and may directly drive adipogenesis and enhance visceral, subcutaneous and ectopic fat deposition (51).

The pathological expansion of white AT in obesity is also associated with an increased release of biologically active adipokines, mainly leptin (52–54). A large number of studies on pubertal development have shown that physiological concentrations of leptin are permissive and promote hypothalamic and pituitary function in males (at central level); on the other hand, if its serum levels exceed a specific threshold, leptin inhibits testicular function (at peripheral level). In fact, *in vitro* studies on Leydig cells have shown that elevated levels of leptin are able to inhibit gonadotropin-mediated testosterone secretion, highlighting a strong correlation between high leptin serum levels, such as those found in obesity, and reduced androgen levels (55). The aforementioned studies allow us to understand what happens in case of leptin resistance, which represents a hallmark of obesity. Leptin resistance (which can be due to either defects in the intracellular signaling of the leptin receptor or to an abnormal leptin transport across the blood–brain barrier) reduces GnRH secretion through a downregulation of kisspeptin gene expression and its receptors (56–58). On the contrary, it seems that leptin sensitivity is maintained in the testis, thus inhibiting steroidogenesis. Therefore, the combination of leptin resistance at hypothalamic-pituitary level and its preserved sensitivity in the testes sustains the hypogonadal state (59). Furthermore, obesity is accompanied by low-grade inflammation and the overproduction of inflammatory mediators (such as IL-6, IL-1, TNF-alpha), and is associated with reduced testosterone levels, suppressing the release of gonadotropin hormones (especially LH), intervening with the translational mechanisms of the GnRH transcript (42).

Oxidative stress, subsequent to the expansion of AT and an insufficient parallel increase in blood flow and oxygen supply, can increase the generation of reactive oxygen species, affecting the steroidogenic pathway in Leydig cells, eventually leading to decreased testosterone production and infertility. In addition, oxidative stress also enhances cortisol secretion, which in turn affects LH secretion, thus reducing testosterone production (60–62). Recently, the role of sirtuins as key metabolic sensors for body homeostasis has emerged: in fact, much evidence has shown their role in obesity and male fertility, not only by being involved in lipid metabolism but also by regulating mechanisms crucial for spermatogenesis, including glycolysis, fatty acids oxidation and oxidative stress. So, regulating sirtuins activity through pharmacological and non-pharmacological intervention could represent a novel treatment for obesity related FHH (63). Lifestyle interventions are the rationale of treatment for individuals with obesity. In fact, weight loss of at least 10% contributes to significantly raise circulating testosterone levels and improves symptoms related to androgen deficiency (8, 10). In particular, the effects of the very low-calorie ketogenic diet seem promising to improve insulin-resistance and β-cell dysfunction, thus ameliorating testosterone levels and gonadal function, as described in novel studies (64, 65). However, diet and behavioral therapies have an elevated rate of failure in the long-term, therefore different weight loss drugs have been developed, and several more are currently under investigation. In a recent study, patients with metabolic hypogonadism, treated with liraglutide for 4 months, showed an improvement in sexual function and sperm parameters compared to those treated with gonadotropins or testosterone, suggesting that GLP1-analogs can be useful in weight reduction and also testicular function recovery (66). Bariatric surgery is indicated as the ultimate approach, despite being able to significantly increase the secretion of testosterone (67, 68).

TRT could be useful in promoting fat mass loss and improving metabolic outcomes through the direct effects on AT function in hypogonadal subjects. Several studies have shown that TRT causes a significant decrease in body weight and waist circumference. In addition, as stated by recent meta-analysis, TRT promotes the reduction of fat mass (particularly in the visceral compartment) and the increase of lean mass in men with testosterone deficiency (12, 69–71).

## Drugs

Several commonly used drugs are associated with secondary FHH in men. Opioids are widely employed for pain management, as well as for detoxification of opioid addiction. They act on μ-opioid receptors in the hypothalamus and pituitary gland, leading to a decreased pulsatile release of GnRH, reduced LH (as well as FSH) pulse frequency, and ultimately to reduced sex hormone levels. The prevalence of male hypogonadism is estimated between 19 and 86% (72), and is influenced by treatment duration, dosage and specific opioid medication (73, 74). Prolonged use of opioids causes symptomatic androgen deficiency, resulting in sexual dysfunction and impaired sperm production. The most prominent abnormality on semen analysis is asthenozoospermia, followed by teratozoospermia and oligozoospermia (75). Functional δ-, κ- and μ-opioid receptors are also present on human spermatozoa, and opioids may also have independent direct effects on spermatogenesis (76). Opioids can also increase prolactin levels, thereby further reducing testosterone levels (77, 78). Opioid withdrawal is typically followed by a recovery in serum testosterone levels within one month (72). The benefits and risks of TRT in the context of opioid-induced hypogonadism have not been thoroughly evaluated so far (2). TRT should be considered in hypogonadism due to chronic use of opioids, whereas short-term use does not require treatment. TRT has been associated with improved sexual function (79) and body composition, without significant changes in pain perception and quality of life (80).

Sex steroids suppress the HPG-A and their chronic administration may cause androgen deficiency and infertility. The (mis)use of testosterone is particularly common among athletes and bodybuilders to enhance physical performances or muscle mass (81, 82). Chronic use leads to suppression of gonadotropin levels, as well as testicular hypotrophy and oligozoospermia or azoospermia (83). Androgen deficiency symptoms during use are typically absent. After their discontinuation the HPG-A recovers within weeks to months, however, a sustained suppression can last up to years in a limited number of subjects (84). Prolonged hypogonadism can be associated with sexual and mood symptoms, and might necessitate therapy with low-dose TRT or hCG, although the use of clomiphene citrate or aromatase inhibitors has also been advocated (85).

GnRH analogues are typically used in men with prostate cancer, as well as in children with precocious puberty. Therapy withdrawal determines the reactivation of the HPG-A within weeks to months, although some older men experience longer suppression periods (86, 87).

Long-term use of glucocorticoids can determine exogenous Cushing syndrome, a common cause of secondary hypogonadism, resulting in androgen deficiency and sperm alterations (88, 89). Glucocorticoids suppress GnRH secretion, resulting in low-normal gonadotropin levels and suppressed testosterone concentrations. Prednisone-equivalent doses as low as 5 mg daily can cause hypogonadism in older men. Given the reduced SHBG concentrations, the evaluation of free testosterone levels is mandatory in this context. The use of TRT has been associated with improved body composition, bone mineral density, and quality of life (90).

## HIV/AIDS

Highly-active antiretroviral therapy (HAART) has greatly changed the natural history of HIV infection, turning it into a chronic disease with several comorbidities that can also involve the endocrine system (91). In this respect, there is a long-lasting association between HIV and FHH, especially in the pre-HAART era, mostly due to advanced immunosuppression and opportunistic infections in AIDS. Although the introduction of HAART has reduced the incidence of hypogonadism among HIV-infected men, it is still considered the most common endocrine dysfunction, with a prevalence of up to 30% (11, 92). Moreover, this condition is increasingly affecting even adolescents, representing a potential problem for the proper development of testicular function (93).

In HIV-infected men, hypogonadism is caused by an impairment of the HPG-A, resulting in secondary hypogonadism rather than primitive testicular failure. However, very frequently an increase in SHBG levels can coexist in HIV-infected men, such that the determination of free testosterone is of paramount importance, as total testosterone levels may not reveal the condition of FHH (94, 95).

To date, it is still not yet fully understood how HIV leads to the impairment of the HPG-A resulting in FHH. Many causes are called into question, and specifically poor general health and acute illness may lead to a hypogonadal state, as observed in systemic disorders. Furthermore, drugs (especially opiates and HAART *per se*) or opportunistic infections involving the hypothalamic-pituitary area, may have direct effects on the HPG-A, leading to its suppression. HIV- and AIDS-related lipodystrophy and wasting, characterized by changes in body fat distribution (including fat loss - lipoatrophy -, skeletal muscle loss and visceral fat accumulation) can also inhibit gonadotropin secretion through an increased aromatization of androgens to estradiol, in turn inhibiting the HPG-A (96, 97).

A significant alteration of seminal parameters can be observed, specifically oligozoospermia and asthenozoospermia, which are secondary to HAART and reduced testosterone levels. Once FHH has been recognized, and before starting TRT, it is mandatory to assess the desire for fatherhood, and if so, to consider the use of gonadotropins to stimulate spermatogenesis, and/or the referral for assisted reproduction technologies (ART) (98, 99).

Appropriate TRT aims to improve clinical symptoms, such as libido and weight loss, but also to promote weight gain and to increase lean body mass and bone mineral density. Several options (transdermal and intramuscular injections) can be discussed, depending on the severity of hypogonadism and on patient preference (100). In addition, TRT should be administered in HIV patients with reduced body weight and low testosterone levels, in order to maintain weight and lean mass, with an overall improvement in quality of life (36).

## Low energy availability

Acquired FHH may be secondary to a LEA state, deriving from an impaired balance between dietary caloric intake and excessive energy expenditure (101). This condition was first coined for women affected by the “female athletes’ triad” (comprising caloric deficit, menstrual disturbances, and low bone mineral density) (102). In the last decade, the need to also classify male athletes in this context has led to the definition of a broader label, defined as “relative energy deficit-in sport” (103, 104). The energy deficit may be absolute, with a reduced body weight, or relative, with an increase of muscle mass compared to fat mass (105). The underlying pathophysiological mechanism is characterized by the HPG-A impairment, with altered GnRH and LH pulsatility and low testosterone levels, leading to HH (7, 106).

Furthermore, higher prolactin levels and the hyperactivation of the hypothalamic-pituitary-adrenal axis could also be involved in perturbations of the HPG-A in athletes (5, 6). Specifically, psychological stresses can also alter the physiological axis pulsatility either through neuroendocrine circuits or by HPG-A alterations (107, 108).

Leptin, an adipokine involved in the neuroendocrine mechanism underlying the development of HH, plays a central role in signaling available energy stores in adipose tissue, by acting on the arcuate nucleus of the hypothalamus. Reduced levels occur when conditions of energy deficit are present, as a possible consequence of an inadequate nutritional status (109).

Specifically, it modulates GnRH secretion both directly by inhibiting agouti-related peptide and neuropeptide Y (AgRP) and Neuropeptide Y (NPY) neurons and activating pro-opiomelanocortin (POMC) and cocaine and amphetamine-regulated transcript (CART) neurons, and indirectly through kisspeptin neurons (110). Therefore, reduced leptin levels are associated with infertility due to the starvation response that ensures the maintenance of reproductive function only if an adequate energy intake is available (111–113). Finally, as a consequence of HPG-A disruption, decreased libido and altered spermatogenesis may be detected (114, 115).

FHH in LEA is usually reversed by restoring adequate energy intake, correcting the caloric deficit or excessive exercise, and by achieving and maintaining normal body weight (116, 117). However, treatment must consider the etiologic framework: understanding whether disordered eating, eating disorders (EDs), perfectionism, or compulsivity personality traits are at the origin of the excessive exercise should guide in choosing the most appropriate management, which almost necessarily should be combined with a nutritional intervention (104, 118, 119). Athletes in energy deficit may not have a psychological component and may only need education to increase energy intake or reduce excessive expenditure, but these conditions can coexist and partially overlap with EDs, considering the high rates of EDs in athletes (9). In this condition, the use of TRT or other medications to stimulate the HPG-A are not recommended, due to both limited evidence of benefit and potential procrastination in addressing the underlying problem (105, 120). Furthermore, these treatments are banned by the World Anti-Doping Agency for competitive athletes (121). Therefore, the only acknowledged strategies in this context are non-pharmacological: nutritional counseling, modifications in training volume, cognitive-behavioral therapy or other psychotherapies (122, 123). Hence the commitment of the scientific community is needed to develop intervention studies targeted at FHH, including clinical trials, to develop specific treatment guidelines and allow a more comprehensive approach to prevention or early detection of these disorders.s

## Conclusions

FHH represents one of the most relevant causes of testosterone deficiency in men, resulting from extremely frequent conditions, such as obesity and T2DM, as well as LEA, HIV, and drug use. The current idea of FHH has replaced the old concept of late-onset hypogonadism, which revolved around ageing as the cause of hypogonadism. Diagnosis and management require a multidisciplinary approach, starting from the potential resolution of the underlying cause, lifestyle modifications, and consideration of TRT when necessary. As prevalence continues to rise even in young adults, obesity and T2DM are becoming an ever-growing worldwide phenomenon, thus leading to more complex implications in terms of hypogonadism. Similarly, some behaviors such as physical exercise and dietary precautions can become unhealthy and pathological when excessively strict or intense. Likewise, thanks to the innovative available therapies, HIV-infected men live longer now, and chronic related comorbidities are easier to deal with. Likewise young HIV-infected men are also at high risk of developing FHH. Moreover, in most forms of FHH the presence of a two-way causal relationship between testosterone and metabolic disorders has been overtly proven, even if only few interventional studies demonstrate that TRT in hypogonadal men might improve body composition and insulin sensitivity. Conversely, improvement of metabolic disorders, especially interventions aimed at reducing body weight and insulin resistance, eventually lead to an improvement of testosterone levels and HPG-A function. Nonetheless, future interventional studies are needed to elucidate the independent pathogenic role of testosterone deficiency in metabolic disorders, in the FHH setting, and to better establish the possible health effects of TRT in FHH and related comorbidities.

## Author contributions

All the authors have made a substantial, direct and intellectual contribution to the work. MS revised it critically. All authors approved the work for publication.
